# A Novel Municipal-Level Approach to Uncover the Hidden Burden of Hepatitis C: A Replicable Model for National Elimination Strategies

**DOI:** 10.3390/v17101392

**Published:** 2025-10-19

**Authors:** Pietro Torre, Silvana Mirella Aliberti, Tommaso Sarcina, Mariano Festa, Chiara D’Amore, Giuseppe D’Adamo, Michele Gambardella, Antonella Santonicola, Gaetano Manzi, Mario Masarone, Mario Capunzo, Marcello Persico

**Affiliations:** 1 Internal Medicine and Hepatology Unit, Department of Medicine, Surgery and Dentistry, Scuola Medica Salernitana, University of Salerno, 84131 Salerno, Italy; ptorre@unisa.it (P.T.); tsarcina@unisa.it (T.S.); mafesta@unisa.it (M.F.); mmasarone@unisa.it (M.M.); 2 Department of Medicine, Surgery and Dentistry, Scuola Medica Salernitana, University of Salerno, Baronissi, 84081 Salerno, Italy; sialiberti@unisa.it (S.M.A.); mcapunzo@unisa.it (M.C.); 3 Infectious Diseases Unit, Department of Medicine, Surgery and Dentistry, Scuola Medica Salernitana, University of Salerno, 84131 Salerno, Italy; chr.damore@gmail.com (C.D.); g.manzi12@studenti.unisa.it (G.M.); 4 Internal Medicine Unit, Umberto I Hospital, Nocera Inferiore, 84014 Salerno, Italy; g.dadamo@aslsalerno.it; 5 Infectious Diseases Unit, San Luca Hospital, Vallo della Lucania, 84078 Salerno, Italy; gambardella1960@gmail.com; 6 Gastrointestinal Unit, Department of Medicine, Surgery and Dentistry, Scuola Medica Salernitana, University of Salerno, 84131 Salerno, Italy; asantonicola@unisa.it

**Keywords:** hepatitis C, elimination of viral hepatitis, HCV elimination, hidden burden of hepatitis C, HCV epidemiology

## Abstract

Background: Hepatitis C Virus (HCV) remains a global health challenge as WHO elimination targets are not achievable in most countries, mainly due to the high number of undiagnosed individuals. In Italy, where national elimination efforts are ongoing, regional disparities further hinder progress. This study aimed to characterize the hidden burden of chronic HCV infection across t he territory of the Province of Salerno, Southern Italy, to suggest a novel municipal-level screening approach, with implications for national strategies. Methods: We analyzed records of residents diagnosed with chronic HCV infection and linked to care between 2015 and 2022. Data included age, sex, municipality of residence, HCV genotype, and fibrosis stage. Observed prevalence was compared with expected prevalence derived from national/regional benchmarks. Municipalities were categorized as urban or rural based on the resident population. Results: A total of 3528 cases were identified across 139 municipalities. Patients had a mean age of 63 years, and 54% were male. Half were diagnosed at an advanced stage (F3–F4), with genotype 1b being predominant. The hidden burden increased with age and showed a higher prevalence in rural areas compared to urban ones, with values of about 7 vs. 3 per 1000 inhabitants respectively. Logistic regression analysis identified age, male sex, urban residence, and genotype 1b as factors associated with advanced fibrosis or cirrhosis. Conclusions: This is the first Italian study to apply a standardized municipal-level classification to quantify the hidden burden of HCV. The model identifies underdiagnosed areas, highlights urban–rural disparities (a higher degree of underdiagnosis in rural areas versus a higher frequency of late diagnosis in urban ones), and provides a replicable tool for precision public health. Its adoption could enhance national HCV elimination efforts by supporting targeted screening, optimized resource allocation, and equitable access to care.

## 1. Introduction

Although major advances in treatment have made the infection by hepatitis C virus (HCV) curable, it continues to pose a significant global health challenge. Nearly a decade after the World Health Organization (WHO) set the goal of eliminating viral hepatitis by 2030, including hepatitis C, only a limited number of countries are currently on track to achieve this objective, and it is estimated that 50 million individuals are still chronically infected worldwide. Key barriers to elimination include disparities in access to diagnosis and treatment, along with the significant financial and infrastructural burden associated with delivering comprehensive care services [1,2,3,4,5]. Notably, Italy, which has one of the largest populations of HCV-infected subjects in Europe [6], is also among the nations where the impact of such infection appears insufficiently managed according to the Polaris Observatory [7]. Once considered virtuous in the race toward elimination, thanks to its treatment policies and a growing number of treated patients [8,9], the country has subsequently entered a phase of stagnation. This is because the number of patients diagnosed and receiving antiviral treatment in recent years has not been deemed high enough to achieve the established targets [10]. Regional variations in the implementation of hepatitis C-related activities further hinder national progress.

The observed reduction in antiviral therapies over recent years, coupled with an estimated high number of undiagnosed individuals, the so-called “hidden burden” of infections, many of which are associated with advanced liver disease, highlights the crucial importance of screening activities [10,11,12]. Since universal screening is not currently implemented in most countries, the effectiveness of existing strategies depends heavily on the prioritization of populations with a higher expected prevalence. These, according to the “micro-elimination” model, include high-risk subjects such as people who inject drugs (PWID), prisoners, men who have sex with men (MSM), and individuals born within specific age cohorts (e.g., 1945–1965), regardless of knowledge of known risk exposures [2,10,13,14,15]. This approach has a major limitation: it overlooks the role of geographic disparities, failing to capture the healthcare inequalities that penalize hepatitis C care in remote or underserved areas, even in high-income countries. It is well known that remote communities have fewer liver specialists and HCV-related services, with distance being a barrier to accessing them, and with general practitioners (GPs) often representing the only easily accessible point of contact for healthcare. While some countries have improved access to care, for example, by allowing non-specialists to prescribe direct-acting antivirals (DAAs), this is not yet a widespread practice and is not even adopted in Italy [2,16,17,18,19]. In these cases, specific dynamics between local physicians and the community may not ensure equitable access to HCV diagnosis and treatment.

Given the lack of data on these aspects in Italy, we conducted an analysis of the geographic distribution of actively infected HCV patients who underwent antiviral therapy at hepatology centers in the Province of Salerno, as a proxy for individuals who were successfully screened, diagnosed, and linked to care. Particular attention was given to urban–rural differences within the Province, which, in its nearly 5000 km^2^, is home to over a million people, and is located in Campania (southern Italy), an area historically known for high hepatitis C prevalence [20]. By integrating local data with regional and national prevalence benchmarks, we aimed to geographically characterize the hidden burden of HCV, hypothesizing significant disparities between rural and urban areas. The gap which we intended to investigate is not just a mere epidemiological issue; it conceals cases that could progress to cirrhosis, liver decompensation, or hepatocellular carcinoma—conditions that heavily impact a person’s life and the costs of the healthcare system.

The primary objective of this study was to estimate the hidden burden of chronic HCV infection across the Province of Salerno (Campania region), Southern Italy, by applying a novel municipal-level epidemiological model. This was achieved by comparing observed and expected prevalence, derived from regional and national benchmarks, with particular attention to urban–rural disparities, and with the goal of providing population-based evidence supporting national HCV elimination initiatives through identification of priority areas for screening and linkage to care.

In parallel, secondary objectives included describing the demographic and clinical characteristics of chronically HCV-infected people residing in this territory; calculating observed HCV prevalence (diagnosed and linked-to-care cases) at both municipal and provincial levels, and stratified by age group and urban–rural status; estimating the expected prevalence by applying age-specific prevalence rates from the literature to official ISTAT population data, thereby quantifying the hidden burden by age group and geographic setting; supporting a refined classification of municipalities based on discrepancy between observed and expected cases to inform geographically targeted public health strategies.

Our model could offer a replicable framework for regional health authorities to prioritize screening and resource allocation, with potential applicability to other infectious or chronic diseases characterized by hidden prevalence. To the best of our knowledge, this is the first study in Italy to integrate municipal-level prevalence mapping with hidden burden estimates and a novel urban–rural classification system.

## 2. Patients and Methods

### 2.1. Study Design, Inclusion Criteria, and Setting

This is a multicenter, retrospective study aimed at quantifying both the observed and hidden burden of HCV infection in the Province of Salerno, Southern Italy, with the goal of supporting future screening initiatives. The study included patients who had been diagnosed with and treated for chronic hepatitis C, defined by detectable HCV-RNA in serum, and with registered residence in the Province of Salerno during the study period (March 2015–December 2022). Patients treated for hepatitis C at participating centers but living outside the province were excluded from the analysis. Additionally, five patients were excluded because their municipality of residence was unknown. All cases of active infection were included, regardless of clinical status, treatment outcome, or subsequent death. Treatment-related data (e.g., sustained virologic response, SVR) were not collected, as among the study’s objectives was not to evaluate treatment efficacy but rather to measure the cumulative diagnosed and linked-to-care HCV cases. To ensure internal validity and avoid double-counting, records were deduplicated across hospitals by cross-checking personal identifiers available at the extraction stage (name, date of birth, municipality of residence). This procedure was performed before anonymization. After deduplication, all direct identifiers were removed, and only anonymized records were retained for analysis.

Among the 158 municipalities of the province, 139 had at least one confirmed case, while in the remaining 19, no diagnosed and linked-to-care HCV cases were found. These 19 municipalities are predominantly small, all with fewer than 5000 residents, and most are located in peripheral or hilly areas of the province. For the main analysis, a cumulative approach was adopted, aggregating all diagnosed cases identified between 2015 and 2022 without stratification by year of diagnosis. This methodology was selected to estimate period-prevalence, allowing for a comprehensive assessment of the total burden of infections and for the identification of geographic and demographic gaps in case detection; in fact, the study’s objective was to estimate the overall diagnosed and hidden burden at the municipal level rather than changes over time. This strategy ensured sufficient statistical power for smaller municipalities and strengthened the robustness of municipal-level comparisons. However, we also analyzed temporal dynamics by stratifying data on observed cases, hidden burden, and patient characteristics across two 4-year periods, 2015–2018 and 2019–2022.

### 2.2. Data Sources

#### 2.2.1. Clinical Data

Clinical data of HCV cases were extracted from multiple clinical sources, including electronic health records from public healthcare facilities in the Campania region. The dataset included anonymized records of patients diagnosed and linked-to-care for antiviral therapy at major provincial hospitals, which included all five adult DAAs prescribing centers active in the province during the study period: San Giovanni di Dio e Ruggi d’Aragona Hospital (Gastroenterology Unit, Infectious Disease Unit, and Internal Medicine and Hepatology Unit) in Salerno, San Luca Hospital (Infectious Disease Unit) in Vallo della Lucania, and Umberto I Hospital (General Medicine Unit) in Nocera Inferiore.

Data extraction was conducted in 2024 to ensure temporal consistency across sources. The collected variables included age at presentation for therapy, sex, municipality of residence, HCV genotype, and liver fibrosis stage, assessed before treatment with the METAVIR scoring system (F0–F4). Fibrosis was primarily evaluated via vibration-controlled transient elastography (VCTE) (FibroScan^®^, Echosens, Paris, France), with 10 kPa and 13 kPa as cutoffs for advanced fibrosis (F3) or cirrhosis (F4), respectively [21].

#### 2.2.2. Population Data

Municipality-level and age-stratified population data for the Province of Salerno were obtained from the Italian National Institute of Statistics (ISTAT) [22], with reference date 1 January 2025. These data were used as denominators for the calculation of crude, age-specific, and geographically stratified prevalence rates. Population estimates were based on the most recent and official demographic data available at the time of analysis. Given the modest but steady annual population decline observed in the area [23], demographic shifts over the eight-year study period (2015–2022) were considered negligible for prevalence calculations and unlikely to significantly bias the results.

### 2.3. Age Stratification

Both diagnosed cases and the general population were stratified into four age groups, based on ISTAT categories, but adapted to reflect HCV epidemiological patterns [24,25]:

- 0–29 years: younger individuals (recent transmission routes, e.g., injection drug use).
- 30–45 years: younger adults with intermediate risk profiles.
- 46–56 years: middle-aged adults.
- >56 years: older adults, more likely exposed to historical iatrogenic sources (e.g., unscreened blood transfusions and non-sterile procedures).

### 2.4. Urban/Rural Classification

Municipalities were classified as urban or rural based on a threshold of 5000 inhabitants and on population density, using a cutoff of 150 inhabitants/km^2^, in line with ISTAT’s municipal classification methodology and OECD-Eurostat criteria [26,27]. Municipalities with at least 5000 inhabitants and 150 inhabitants/km^2^ were classified as urban. Conversely, municipalities with less than 5000 inhabitants and/or less than 150 inhabitants/km^2^ were classified as rural. This classification aligns with the Eurostat typology: urban areas corresponded to levels 1 and 2 (densely and intermediate populated areas), while rural areas aligned with level 3 (thinly populated areas) [26].

- Urban areas (≥5000 inhabitants and ≥150 inhabitants/km^2^): characterized by higher population density, greater availability of healthcare services (e.g., hospitals, diagnostic centers), and more diversified economic activities. These included 38 municipalities, totaling 791,298 inhabitants.
- Rural areas (<5000 inhabitants and/or <150 inhabitants/km^2^): characterized by lower population density, economies based primarily on agriculture or tourism, and reduced access to specialist healthcare services. These comprised 101 municipalities, totaling 247,430 inhabitants.

### 2.5. Prevalence of Infection and Hidden Burden Estimation

#### 2.5.1. Observed Prevalence

The observed prevalence of chronic HCV infection was calculated for each municipality and for the Province of Salerno overall, using the following formula:

$$Prevalence\;per\;1000=\left(\frac{Number\;of\;observed\;cases}{Population}\right)\times \;1000$$

Prevalence was computed both at the municipal level (e.g., Ottati: 1.73 per 1000) and aggregated by urban and rural areas. Age-specific prevalence was calculated using the following formula:

$${Age-specific\;prevalence}_{i}=\left(\frac{Observed\;cases\;in\;age\;group\;i}{Population\;in\;age\;group\;i}\right)\times \;1000$$

where *i* refers to each of the four predefined age groups (0–29, 30–45, 46–56, and >56 years).

#### 2.5.2. Expected Prevalence of Active HCV Infection

Based on published estimates of the burden of chronic HCV infection in Italy, we used age-specific prevalence rates from national and regional studies as external reference values. At the national level, the overall prevalence of active HCV infection has been estimated at 0.66% (95% CI: 0.66–0.67, as of January 2021), with higher values reported in Southern regions (0.72%) compared to Northern ones (0.54%) [12]. These data were obtained from a mathematical model incorporating variables such as new infections, migrations, and demographic factors (e.g., new births, deaths), with previously treated individuals being subtracted from the estimate. Regional analyses reported a broader range (0.1–2.4%) and demonstrated a clear age-related gradient [20,28,29,30]. From these data, we applied the following age-specific expected prevalences: 0–29 years: 0.12%; 30–45 years: 0.6%; 46–56 years: 1.2%; >56 years: 2.5%. These values, derived from prior epidemiological evidence, reflect reduced transmission in younger cohorts (due to improved medical practices, such as blood screening and single-use needles, and the introduction of DAAs) and higher prevalence in older individuals, who were more likely to have been exposed to historical iatrogenic routes of transmission. The validity of the lower prevalence for younger groups is further supported by SEIEVA surveillance data on acute HCV infections.

For geographic comparisons, expected prevalence ranges were stratified by setting to reflect contextual differences: urban areas 0.6–0.8%, characterized by greater availability of healthcare infrastructure and screening opportunities; and rural areas 0.8–1.0%, typically older in demographic profile and with fewer healthcare facilities and liver specialists, lower public awareness, and potential socioeconomic vulnerabilities [31,32,33]. These reference values were multiplied by ISTAT population estimates to calculate the expected number of HCV cases at municipal and provincial levels. The expected cases were also estimated by assuming the same prevalence (0.6–0.8%) in the two different settings.

#### 2.5.3. Hidden Burden of HCV

The hidden burden was calculated by subtracting the number of observed cases from the expected cases:

Hidden Burden=Expected Cases−Observed Cases

This was performed across municipalities, urban/rural classification, and provincial levels using the expected prevalence rates described above.

#### 2.5.4. Bayesian Prevalence

Building on the cumulative approach aggregating data from 2015 to 2022 described in Section 2.1, Bayesian smoothing was applied as an alternative method to stabilize prevalence rates in municipalities with small populations and low case counts. This approach adjusts raw municipal prevalence estimates by incorporating information from a prior rate, with the degree of shrinkage determined by the population size of each municipality. Observed HCV cases, resident population, and prior rates (7 per 1000 for urban areas, 9 per 1000 for rural areas) were used as inputs. This procedure reduces random variability, yielding more reliable estimates while preserving local differences. A stabilization parameter (τ = 10,000) was used to adjust the degree of shrinkage. Bayesian Prevalence per 1000 was calculated using the formula:

$$Bayesian\;Prevalence=\;\frac{\left(Observed\;Cases+Prior\;Rate\times Population/1000\right)}{Population+\tau /10}\;\times \;1000$$

### 2.6. Municipality Classification Criteria

Municipalities were classified into three epidemiological categories based on Poisson test results, comparing observed to expected case counts:

- Underdiagnosed areas: Observed cases significantly below expected (*p* < 0.05), suggesting gaps in detection or healthcare access.
- Hotspot areas: Observed cases significantly exceeded expected values (*p* < 0.05), indicating possible local transmission clusters or enhanced screening programs.
- Other area: No significant difference (*p* ≥ 0.05) between observed and expected cases.

### 2.7. Statistical Analysis

Descriptive statistics summarized all demographic and clinical variables, and patients were classified according to municipality of residence (urban/rural), age group, sex, HCV genotype, and fibrosis stage. Data are represented as absolute numbers and percentages or mean ± standard deviation (SD). Chi-square test and Mann–Whitney test were used for categorical variables and continuous variables, respectively; multivariate logistic regression was used to identify predictors of F3–F4 fibrosis. The Poisson test was used to evaluate deviations from expected prevalence at the municipality level. Statistical significance was set at *p* < 0.05 (two-tailed). Statistical analysis was conducted using STATA v16.1 and GraphPad Prism 10.4.2.

### 2.8. Ethical Considerations

The study was conducted in accordance with the Declaration of Helsinki and Italian data regulations. All patient data were anonymized and analyzed in aggregate form. Due to the retrospective design, the exclusive use of anonymized records, and publicly available data, formal ethical approval was not required.

## 3. Results

### 3.1. Study Population and Demographics

Between 2015 and 2022, a total of 3528 confirmed and linked-to-care chronic active HCV cases were identified across 139 municipalities in the Province of Salerno, covering 98.5% of the provincial population (1,038,728) (Table 1). Urban areas comprised 38 municipalities (791,298 inhabitants), accounting for 86.9% (3067) of cases, while rural areas included 101 municipalities (247,430 inhabitants), with 13.1% (461) of cases. All 19 municipalities with no identified cases were rural, comprising a total population of 16,038 inhabitants.

The age distribution of cases showed a marked predominance in older adults, with the mean age being approximately 63 years. Individuals aged >56 years represented 65.5% of all cases (2310/3528), followed by those aged 46–56 (21.7%), 30–45 (11.7%), and 0–29 (1.1%). A slight male predominance was observed (54.7%), and fibrosis staging revealed that 50.2% of patients were diagnosed at advanced stages (F3–F4), suggesting a significant delay in diagnosis and treatment initiation, meaning, in turn, that many patients were identified only when their risk of serious outcomes was greater. Genotype 1b was the most prevalent (38.6%), followed by genotype 2 (31.3%), genotype 3 (13.3%), genotype 1a (12.0%), genotype 4 (4.6%), and other genotypes (0.3%).

Overall, observed HCV prevalence was 0.34% (3.40 per 1000 population; 95% CI: 0.33–0.35), while the total expected number of HCV cases (using an estimated prevalence of 0.72%) was about 7479, indicating a hidden burden of 3951 undiagnosed cases.

Despite having a smaller estimated total of undiagnosed cases, in rural areas the prevalence of the hidden burden per 1000 inhabitants was more than double compared to urban ones (3.12 in urban vs. 7.14 in rural areas, and slightly higher if we also include the 19 rural municipalities with no detected cases); by using the same expected prevalence of cases (of 0.6–0.8%) in the two settings, rural areas still had a higher prevalence of undiagnosed cases (5.14 per 1000 inhabitants). In particular, urban areas, comprising 791,298 inhabitants, demonstrated a greater number of diagnosed, linked-to-care cases. Nevertheless, a substantial hidden burden of undiagnosed infection persists. Applying an expected prevalence range of 0.6–0.8%, the estimated number of HCV cases in urban areas was 4748–6330, compared to 3067 observed cases. This corresponds to an estimated hidden burden of 1681–3263 undiagnosed individuals. In contrast, rural areas (247,430 inhabitants) exhibited a lower number of cases and observed prevalence of 1.86 per 1000 (461/247,430), compared to urban areas (3.88 per 1000; 3067/791,298; *p* < 0.001 for urban vs. rural prevalence). By using a higher expected prevalence of 0.8–1.0% in this context, the estimated number of cases in rural areas corresponded to 1979–2474, yielding a hidden burden of 1518–2013 undiagnosed cases. These results are summarized in Table 2.

### 3.2. Municipal-Level Analysis and Geographic Classification

Municipal prevalence varied substantially, ranging from 0.03% (0.27 per 1000 in Positano) to 1.26% (12.61 per 1000 in Salento). Table 3 summarizes the classification of municipalities based on the results of the Poisson test:

- Underdiagnosed (UD): Municipalities with significantly fewer observed cases than expected, *p* < 0.05 (observed < expected), indicating high hidden burden (e.g., Acerno, Agropoli, and others).
- Hotspots (HS): Municipalities with significantly more observed cases than expected, *p* < 0.05 (observed > expected), suggesting effective screening or localized outbreaks (e.g., Salento).
- Other (OTH): Municipalities with no significant difference between observed and expected cases, *p* ≥ 0.05. (e.g., Albanella, Atrani, and others).

The application of Bayesian smoothing refined prevalence estimates by stabilizing values through the incorporation of a prior rate. Bayesian prevalence ranged from 0.22 per 1000 (Positano) to 8.02 per 1000 (Salento), mirroring the previously described range. Estimates showed little deviation from observed prevalences in the most populated municipalities, while in municipalities with smaller populations and fewer cases, the values were more pronouncedly smoothed.

**Figure 1:**
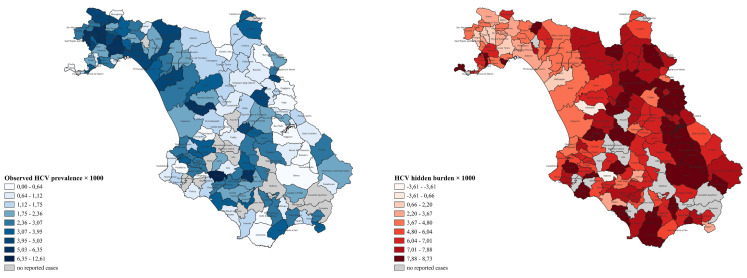
The first map shows the observed HCV prevalence × 1000 residents across the municipalities of the Province of Salerno. The second one displays the HCV hidden burden × 1000 residents, calculated as the ratio between the central value of the hidden burden (presented as a range in Table 3) and the resident population of each municipality, ×1000. These maps highlight a parallel trend: higher numbers of diagnosed and linked-to-care patients are found in areas such as the Agro Nocerino-Sarnese and the city of Salerno, compared to more peripheral regions; conversely, the hidden burden shows an opposite pattern, being higher in these peripheral areas, which also include many rural municipalities with no diagnosed/linked-to-care cases. Consequently, they reflect the study’s key finding: diagnostic coverage is more concentrated in urban centers, while the most significant need for screening exists in peripheral and rural areas. Maps were created using Quantum Geographic Information System (QGIS), version 3.34.8-Prizren, for visualization purpose only.

Figure 1 depicts the observed HCV prevalence and hidden burden in the different municipalities.

### 3.3. Age-Specific Prevalence and Diagnostic Gaps

Age-stratified analysis (Table 4) revealed increasing prevalence and hidden burden with age (with the highest values in the >56 age group, in which ~25% of the cases have been diagnosed), and with significant underdiagnosis across all groups (*p* < 0.001):

### 3.4. Multivariate Predictors of Advanced Liver Disease (F3–F4 Fibrosis)

A multivariable logistic regression model was applied to the full patient cohort (n = 3528) to identify independent predictors of F3–F4 fibrosis, adjusting for age, sex, residential context (urban vs. rural), and HCV genotype. Full results are reported in Table 5.

Age emerged as the strongest predictor of F3–F4 fibrosis, with a stepwise increase in risk:

- Patients aged ≥56 years had more than fourfold increased odds compared to those aged 0–29 years, OR = 4.50; 95% CI: 2.14–10.6; *p* <0.001.
- Those aged 46–56 years had an OR of 2.93 (95% CI: 1.38–6.95; *p* = 0.008).
- Those aged 30–45 years had an OR of 1.28 (95% CI: 0.590–3.07; *p* = 0.56).

Notably, urban residence was associated with higher likelihood of being diagnosed with advanced fibrosis or cirrhosis compared to rural areas (OR = 1.29; 95% CI: 1.05–1.58; *p* = 0.01), as well as infection by genotype 1b (OR = 1.56; 95% CI: 1.35–1.80; *p* < 0.001) and male sex (OR = 1.63; 95% CI: 1.42–1.89; *p* < 0.001).

### 3.5. Temporal Analysis

The temporal analysis (Table 6) documented a reduction in the number of observed and linked-to-care cases between the two periods considered, in line with regional and national reports, with a lower reduction in the burden in the 2019–2022 period compared to 2015–2018 (of 24.6% and 30.0% respectively). In parallel, there was a change in patient characteristics, with a decrease in the mean age and in the percentage of patients presenting for treatment with F3–F4 fibrosis, likely due to the expansion of treatment criteria. Conversely, there was no significant difference in the percentage of patients from the two different settings (urban vs. rural) across the two time periods.

## 4. Discussion

Despite the widespread availability of the DAAs, HCV infection continues to represent a silent yet persistent public health challenge in Italy and worldwide. A substantial proportion of the disease burden remains unseen due to the significant number of undiagnosed individuals, particularly among older adults and residents of geographically or socioeconomically marginalized communities. Against this backdrop, the present study aimed to offer a novel epidemiological analysis, trying to characterize the hidden burden of HCV infection, through the integration of the evaluation of observed and expected prevalence, official demographic data, and a new urban–rural classification system. This was applied to the vast territory of the province of Salerno, in Campania (Southern Italy), a region consistently affected by hepatitis C based on historical data. This framework could provide regional health authorities with a replicable, data-driven tool to guide the strategic allocation of screening and treatment resources, prioritizing specific risk areas, also in other regions. While previous Italian studies have examined national or regional prevalence and associated risk factors [20,28,34,35], none have achieved the level of spatial and demographic resolution or examined the hidden burden and differences between urban and rural areas. We also calculated Bayesian prevalence to improve estimate accuracy, especially by smoothing values in municipalities with small populations and low case counts.

Our results highlight a significant hidden burden of active HCV infection in the territory of Salerno (approximately 4000 people), with a complex relationship between urban and rural areas: urban areas show a higher number of cases and hidden burden in absolute terms, explained by the higher number of residents, despite a lower prevalence of estimated undiagnosed cases compared to rural areas. Our urban–rural classification system allowed quantification of such disparities that were previously invisible in aggregated regional data. This disparity is likely attributable to factors such as a lack of healthcare infrastructure, less frequent use of diagnostic tools, and longer travel distances to services in rural areas. It could also be due to a different distribution of risk factors in the two different settings, such as a historically greater injecting drug use (IDU) in urban areas. In addition to the comparison of undiagnosed/untreated cases, this subdivision reveals other relevant aspects. For instance, our data indicate that individuals in urban areas were more frequently diagnosed with more advanced liver disease (F3–F4 fibrosis). This may reflect greater disease awareness and access to diagnostic services, resulting in greater detection of advanced disease compared to rural areas, potentially, on the other hand, at greater risk of diagnostic delays. Alternatively, the influence of favorable environmental and sociocultural factors that delay clinical manifestation could intervene, linking the increased burden of more severe liver disease also to environmental stressors, known to adversely affect hepatic and immune functions. In fact, it is known that longevity in Cilento, a large area in the central–southern part of the Province of Salerno, is associated with favorable environmental factors [36,37,38], which could contribute to improved immune-metabolic resilience and lower systemic and liver inflammation. This could translate into a lower prevalence of metabolic dysfunction-associated steatotic liver disease (MASLD) and its associated cardiometabolic risk factors (like obesity and diabetes) in some areas of this region, thereby potentially slowing or reducing HCV-related fibrogenesis [39,40]. The different prevalence of MASLD between rural/urban contexts due to different lifestyles, contributing to a different manifestation of HCV-related disease and translating into fewer cases of advanced fibrosis or cirrhosis in the rural areas of Cilento, remains a hypothesis to be confirmed in further studies. However, this interpretation is coherent with prior research in longevity zones like Cilento, where protective environmental factors (including lifestyle) may delay the progression or expression of chronic diseases [41,42]. While speculative, this perspective aligns with our findings, and it cannot be excluded that both biological protection and diagnostic vulnerability coexist, though further research is needed to substantiate these claims.

Regarding age, beyond documenting more HCV-infected, linked-to-care individuals in older age groups, our study additionally highlights an age-dependent rise in hidden hepatitis C infections. When considered alongside recent projects from northern and southern Italy, these results underscore the imperative to broaden free HCV screening eligibility to older people, coupled with greater involvement of GPs and non-liver specialists (e.g., through opportunistic screening), a measure that aligns with appeals from the Italian hepatological community to the government [11,29,43,44,45]. By limiting it to the 1969–1989 birth cohort (the age group in which free screening is currently provided in Italy in the general population setting), we are currently missing crucial opportunities for early detection and intervention. In fact, older age is generally associated with a longer disease duration, which is linked to a higher risk of advanced fibrosis. This is well-documented in the literature and further corroborated by the association we found in our cohort among older age, genotype 1b (the most common genotype in the general population in Italy), and more severe liver disease [11].

Data analysis between two distinct timeframes, 2015–2018 and 2019–2022, enabled an examination of temporal trends in the number of patients and their characteristics. The reduction in the number of observed, linked-to-care cases between the two periods aligns with the declining trend registered both in Italy and globally [3,46], and is associated with a smaller decline in hidden burden in the later period. Concurrently, the reduction in the mean age and the proportion of patients presenting for treatment with F3–F4 fibrosis can be linked to the broadening of treatment eligibility criteria. This latter finding is consistent with nationwide trends; however, over the years following the expansion of the criteria (introduced in 2017), there was not a significant decline in late diagnoses and treatment start, which again underscores the need for sustained interventions [11,47].

Taken together, our results demonstrate the potential of a municipality-level approach to generate precision public health insights. By integrating epidemiological modeling with demographic and spatial data, our study highlights both the determinants of underdiagnosis and the ecological contexts in which they occur, potentially providing a replicable and policy-relevant model to support precision public health strategies. These could involve establishing new prescribing centers in underserved areas or empowering local GPs or non-liver specialists to manage hepatitis C, guided by liver specialists (task-sharing), as is already the case in some countries on track for elimination. This guidance could potentially be provided through telemedicine (like the Extension for Community Healthcare Outcomes, ECHO, model). Alternatively, time-limited screening campaigns, also utilizing temporary facilities or mobile units—like efforts previously conducted in Egypt—could expand our capacity to identify and treat patients [2,48,49].

This study has several limitations. The analysis focuses on patients diagnosed and treated at specialized centers in the province of Salerno, reflecting linked-to-care cases rather than the full prevalence of active infection, which, aligns with our aim to estimate the hidden burden, however. Moreover, the expected prevalence used to calculate this burden (approximately 4000 subjects) is itself an estimate and therefore inherently uncertain. The municipal-level analysis provides population-level insights, and while it is ecological in nature, caution is advised when applying findings to individual risks. The cumulative approach, combining eight years of data, was a necessary methodological choice to ensure statistical stability and reliable estimates at the municipal level, particularly for small populations. The addition of a temporal analysis comparing two periods (2015–2018 vs. 2019–2022) partially mitigates this by revealing trends in burden and patient characteristics, such as changes in age and disease stage at presentation. However, this temporal assessment is constrained by its broad time intervals, which may obscure shorter-term fluctuations or local variations, limiting its ability to capture dynamic changes fully. Further refinements, such as hierarchical Poisson regression or geostatistical models, could enhance the integration of temporal trends, improve uncertainty quantification, and integrate contextual covariates like socioeconomic indicators or healthcare accessibility, offering a more nuanced understanding of the hidden burden. Lastly, our study does not directly account for high-risk populations such as PWID, MSM, incarcerated individuals, and homeless people, whose distribution is uneven and likely concentrated in urban areas. Due to the absence of reliable, municipal-level epidemiological data for these groups in Italy, our estimates were based primarily on age structure and urban–rural classification. Future adaptations of the model could benefit from integrating data from addiction centers, prisons, or other at-risk contexts, thereby refining its capacity to guide precision public health interventions in both urban and rural settings.

## 5. Conclusions

In conclusion, we observed differences in HCV care and estimated hidden burden across the vast territory of the province of Salerno, with a complex relationship between rural and urban contexts. Through the model we applied, and that can be replicated in other areas and for other diseases, we documented a more than double prevalence of HCV hidden burden in rural areas compared to urban ones, with the latter, conversely, being characterized by a greater probability of diagnosis of advanced fibrosis or cirrhosis. By prioritizing municipalities with the highest diagnostic gaps and adapting interventions to local contexts based on our approach, a transition from broad, non-targeted strategies to precision public health can be implemented. From a policy perspective, we recommend prioritizing underdiagnosed municipalities for immediate, targeted interventions, which may include increased GP-led opportunistic testing, task-sharing models supported by specialist oversight (also based on telemedicine), the involvement of mobile screening units, and time-limited outreach campaigns. These should occur alongside improved data linkages to addiction, correctional, and harm-reduction services. These measures, coupled with periodic reassessment using the proposed framework, could increase case-finding efficiency and accelerate progress toward national and WHO elimination targets.

## Figures and Tables

**Table 1:** Characteristics of the study population.

Characteristic	Value	*p*-Value
Total Provincial Population	1,054,766	-
Population Covered (139 Municipalities)	1,038,728 (98.5% of the province)	-
Population Missing (19 Municipalities)	16,038 (1.5% of the province)	-
Total Confirmed HCV Cases	3528	-
Urban Municipalities	38 (791,298 inhabitants, 3067 cases, 86.9%)	
Rural Municipalities	101 (247,430 inhabitants, 461 cases, 13.1%)	<0.001 *
Age Distribution		
0–29 years	294,967 (38 cases, 1.1%)	
30–45 years	203,548 (414 cases, 11.7%)	
46–56 years	171,274 (766 cases, 21.7%)	
>56 years	368,939 (2310 cases, 65.5%)	<0.001 **
Mean Age (years) ± SD	62.7 (14.1)	-
HCV Genotype	1b (38.6%)	-
	2 (31.3%)	
	3 (13.3%)	
	1a (12.0%)	
	4 (4.6%)	
	Others (0.3%)	
Sex		-
Female	1598 (45.3%)	
Male	1930 (54.7%)	
Disease Stage	1757 (49.8%) F0–F2	-
	1771 (50.2%) F3–F4	
Study Period	2015–2022	-

* Chi-square test for urban vs. rural case distribution. ** Chi-square test for age group case distribution.

**Table 2:** Urban–Rural Disparities in HCV Prevalence.

Parameter	Urban	Rural
Population	791,298	247,430
Observed cases	3067	461
Observed prevalence per 1000	3.88	1.86
Expected prevalence range	0.6–0.8%	0.8–1.0%
Expected cases	4748–6330	1979–2474
Hidden burden (undiagnosed cases)	1681–3263	1518–2013
Prevalence of hidden burden per 1000	3.12	7.14

**Table 3:** Prevalence, Hidden Burden, and Classification for all 139 Municipalities.

MUN	Type	POP	OBS_C	OBS_P	BAY_P	EXP_Min–Max	HB_Min–Max	*p*	CLS
**Agropoli**	Urban	21,283	62	2.91	2.79	127.7–170.3	65.7–108.3	<0.001	UD
**Albanella**	Urban	6311	40	6.34	5.48	37.9–50.5	−2.1–10.5	0.051	OTH
**Angri**	Urban	34,136	205	6.01	5.84	204.8–273.1	−0.2–68.1	0.002	UD
**Ascea**	Urban	5846	21	3.59	3.07	35.1–46.8	14.1–25.8	<0.001	UD
**Baronissi**	Urban	16,859	39	2.31	2.19	101.2–134.9	62.2–95.9	<0.001	UD
**Battipaglia**	Urban	49,395	237	4.80	4.71	296.4–395.2	59.4–158.2	<0.001	UD
**Bellizzi**	Urban	13,299	36	2.71	2.52	79.8–106.4	43.8–70.4	<0.001	UD
**Bracigliano**	Urban	5291	2	0.38	0.32	31.7–42.3	29.7–40.3	<0.001	UD
**Capaccio Paestum**	Urban	22,412	53	2.36	2.27	134.5–179.3	81.5–126.3	<0.001	UD
**Casal Velino**	Urban	5360	8	1.49	1.26	32.2–42.9	24.2–34.9	<0.001	UD
**Castel San Giorgio**	Urban	13,705	31	2.26	2.11	82.2–109.6	51.2–78.6	<0.001	UD
**Castellabate**	Urban	8679	9	1.04	0.94	52.1–69.4	43.1–60.4	<0.001	UD
**Cava de’ Tirreni**	Urban	49,754	273	5.49	5.39	298.5–398.0	25.5–125.0	<0.001	UD
**Eboli**	Urban	37,578	91	2.42	2.37	225.5–300.6	134.5–209.6	<0.001	UD
**Fisciano**	Urban	14,110	36	2.55	2.39	84.7–112.9	48.7–76.9	<0.001	UD
**Maiori**	Urban	5217	16	3.07	2.58	31.3–41.7	15.3–25.7	<0.001	UD
**Mercato San Severino**	Urban	21,451	75	3.50	3.35	128.7–171.6	53.7–96.6	<0.001	UD
**Montecorvino Pugliano**	Urban	11,119	29	2.61	2.40	66.7–89.0	37.7–60.0	<0.001	UD
**Montecorvino Rovella**	Urban	12,286	27	2.20	2.04	73.7–98.3	46.7–71.3	<0.001	UD
**Nocera Inferiore**	Urban	43,424	245	5.64	5.52	260.5–347.4	15.5–102.4	<0.001	UD
**Nocera Superiore**	Urban	23,495	102	4.34	4.17	141.0–188.0	39.0–86.0	<0.001	UD
**Olevano sul Tusciano**	Urban	6608	15	2.27	1.98	39.6–52.9	24.6–37.9	<0.001	UD
**Pagani**	Urban	35,086	196	5.59	5.44	210.5–280.7	14.5–84.7	<0.001	UD
**Pellezzano**	Urban	10,884	39	3.58	3.29	65.3–87.1	26.3–48.1	<0.001	UD
**Pontecagnano Faiano**	Urban	26,581	105	3.95	3.81	159.5–212.6	54.5–107.6	<0.001	UD
**Roccapiemonte**	Urban	8705	29	3.33	2.99	52.2–69.6	23.2–40.6	<0.001	UD
**Sala Consilina**	Urban	12,142	13	1.07	1.00	72.9–97.1	59.9–84.1	<0.001	UD
**Salerno**	Urban	125,958	559	4.44	4.41	755.7–1007.7	196.7–448.7	<0.001	UD
**San Cipriano Picentino**	Urban	6674	15	2.25	1.96	40.0–53.4	25.0–38.4	<0.001	UD
**San Marzano sul Sarno**	Urban	10,192	27	2.65	2.42	61.2–81.5	34.2–54.5	<0.001	UD
**San Valentino Torio**	Urban	10,922	31	2.84	2.61	65.5–87.4	34.5–56.4	<0.001	UD
**Sant’Egidio del Monte Albino**	Urban	7676	26	3.39	3.00	46.1–61.4	20.1–35.4	<0.001	UD
**Sapri**	Urban	6345	23	3.62	3.14	38.1–50.8	15.1–27.8	<0.001	UD
**Sarno**	Urban	30,751	133	4.33	4.20	184.5–246.0	51.5–113.0	<0.001	UD
**Scafati**	Urban	47,706	136	2.85	2.80	286.2–381.6	150.2–245.6	<0.001	UD
**Siano**	Urban	9278	45	4.85	4.38	55.7–74.2	10.7–29.2	0.002	UD
**Vallo della Lucania**	Urban	7835	22	2.81	2.50	47.0–62.7	25.0–40.7	<0.001	UD
**Vietri sul Mare**	Urban	6945	16	2.30	2.02	41.7–55.6	25.7–39.6	<0.001	UD
**Acerno**	Rural	2464	3	1.22	0.87	19.7–24.6	16.7–21.6	<0.001	UD
**Altavilla Silentina**	Rural	7032	15	2.13	1.88	56.3–70.3	41.3–55.3	<0.001	UD
**Amalfi**	Rural	4611	18	3.90	3.22	36.9–46.1	18.9–28.1	<0.001	UD
**Atena Lucana**	Rural	2380	3	1.26	0.89	19.0–23.8	16.0–20.8	<0.001	UD
**Atrani**	Rural	764	3	3.93	1.70	6.1–7.6	3.1–4.6	0.056	OTH
**Auletta**	Rural	2143	2	0.93	0.64	17.1–21.4	15.1–19.4	<0.001	UD
**Bellosguardo**	Rural	679	3	4.42	1.79	5.4–6.8	2.4–3.8	0.084	OTH
**Buccino**	Rural	4473	5	1.12	0.92	35.8–44.7	30.8–39.7	<0.001	UD
**Buonabitacolo**	Rural	2431	1	0.41	0.30	19.4–24.3	18.4–23.3	<0.001	UD
**Caggiano**	Rural	2479	1	0.40	0.29	19.8–24.8	18.8–23.8	<0.001	UD
**Calvanico**	Rural	1399	1	0.71	0.42	11.2–14.0	10.2–13.0	<0.001	UD
**Camerota**	Rural	6774	2	0.30	0.27	54.2–67.7	52.2–65.7	<0.001	UD
**Campagna**	Rural	17,060	25	1.47	1.39	136.5–170.6	111.5–145.6	<0.001	UD
**Campora**	Rural	311	1	3.22	0.76	2.5–3.1	1.5–2.1	0.170	OTH
**Cannalonga**	Rural	945	6	6.35	3.09	7.6–9.5	1.6–3.5	0.106	OTH
**Casalbuono**	Rural	1018	1	0.98	0.50	8.1–10.2	7.1–9.2	<0.001	UD
**Caselle in Pittari**	Rural	1865	4	2.14	1.40	14.9–18.7	10.9–14.7	<0.001	UD
**Castelcivita**	Rural	1383	1	0.72	0.42	11.1–13.8	10.1–12.8	<0.001	UD
**Castelnuovo Cilento**	Rural	2831	7	2.47	1.83	22.6–28.3	15.6–21.3	<0.001	UD
**Castelnuovo di Conza**	Rural	561	2	3.57	1.28	4.5–5.6	2.5–3.6	0.082	OTH
**Castel San Lorenzo**	Rural	2174	1	0.46	0.32	17.4–21.7	16.4–20.7	<0.001	UD
**Celle di Bulgheria**	Rural	1685	1	0.59	0.38	13.5–16.9	12.5–15.9	<0.001	UD
**Centola**	Rural	4954	7	1.41	1.18	39.6–49.5	32.6–42.5	<0.001	UD
**Ceraso**	Rural	2205	4	1.81	1.25	17.6–22.1	13.6–18.1	<0.001	UD
**Cetara**	Rural	1919	2	1.04	0.69	15.4–19.2	13.4–17.2	<0.001	UD
**Cicerale**	Rural	1165	4	3.43	1.85	9.3–11.7	5.3–7.7	0.014	UD
**Colliano**	Rural	3397	5	1.47	1.14	27.2–34.0	22.2–29.0	<0.001	UD
**Conca dei Marini**	Rural	636	2	3.14	1.23	5.1–6.4	3.1–4.4	0.054	OTH
**Contursi Terme**	Rural	3158	4	1.27	0.97	25.3–31.6	21.3–27.6	<0.001	UD
**Controne**	Rural	771	1	1.30	0.57	6.2–7.7	5.2–6.7	0.007	UD
**Corbara**	Rural	2484	11	4.43	3.16	19.9–24.8	8.9–13.8	0.003	UD
**Corleto Monteforte**	Rural	479	1	2.09	0.68	3.8–4.8	2.8–3.8	0.058	OTH
**Cuccaro Vetere**	Rural	511	1	1.96	0.66	4.1–5.1	3.1–4.1	0.046	UD
**Felitto**	Rural	1175	1	0.85	0.46	9.4–11.8	8.4–10.8	<0.001	UD
**Furore**	Rural	680	1	1.47	0.60	5.4–6.8	4.4–5.8	0.013	UD
**Futani**	Rural	1052	4	3.80	1.95	8.4–10.5	4.4–6.5	0.026	UD
**Giffoni Sei Casali**	Rural	4950	10	2.02	1.69	39.6–49.5	29.6–39.5	<0.001	UD
**Giffoni Valle Piana**	Rural	11,456	53	4.63	4.26	91.6–114.6	38.6–61.6	<0.001	UD
**Gioi**	Rural	1072	1	0.93	0.49	8.6–10.7	7.6–9.7	<0.001	UD
**Giungano**	Rural	1284	1	0.78	0.44	10.3–12.8	9.3–11.8	<0.001	UD
**Ispani**	Rural	959	1	1.04	0.51	7.7–9.6	6.7–8.6	0.002	UD
**Laureana Cilento**	Rural	1270	4	3.15	1.77	10.2–12.7	6.2–8.7	0.008	UD
**Laurino**	Rural	1214	3	2.47	1.36	9.7–12.1	6.7–9.1	0.004	UD
**Laurito**	Rural	663	2	3.02	1.21	5.3–6.6	3.3–4.6	0.046	UD
**Laviano**	Rural	1278	4	3.13	1.76	10.2–12.8	6.2–8.8	0.007	UD
**Lustra**	Rural	1014	3	2.96	1.49	8.1–10.1	5.1–7.1	0.014	UD
**Minori**	Rural	2559	9	3.52	2.54	20.5–25.6	11.5–16.6	<0.001	UD
**Moio della Civitella**	Rural	1812	5	2.76	1.78	14.5–18.1	9.5–13.1	<0.001	UD
**Montecorice**	Rural	2579	4	1.55	1.12	20.6–25.8	16.6–21.8	<0.001	UD
**Monte San Giacomo**	Rural	1372	1	0.73	0.43	11.0–13.7	10.0–12.7	<0.001	UD
**Montesano sulla Marcellana**	Rural	6231	12	1.93	1.67	49.8–62.3	37.8–50.3	<0.001	UD
**Novi Velia**	Rural	2349	5	2.13	1.50	18.8–23.5	13.8–18.5	<0.001	UD
**Ogliastro Cilento**	Rural	2291	4	1.75	1.22	18.3–22.9	14.3–18.9	<0.001	UD
**Oliveto Citra**	Rural	3602	2	0.56	0.44	28.8–36.0	26.8–34.0	<0.001	UD
**Omignano**	Rural	1641	1	0.61	0.38	13.1–16.4	12.1–15.4	<0.001	UD
**Orria**	Rural	912	2	2.19	1.05	7.3–9.1	5.3–7.1	0.009	UD
**Ottati**	Rural	579	1	1.73	0.64	4.6–5.8	3.6–4.8	0.028	UD
**Padula**	Rural	4794	12	2.50	2.08	38.4–47.9	26.4–35.9	<0.001	UD
**Palomonte**	Rural	3768	5	1.33	1.06	30.1–37.7	25.1–32.7	<0.001	UD
**Perdifumo**	Rural	1803	1	0.55	0.36	14.4–18.0	13.4–17.0	<0.001	UD
**Pertosa**	Rural	649	1	1.54	0.61	5.2–6.5	4.2–5.5	0.017	UD
**Petina**	Rural	994	5	5.03	2.51	8.0–9.9	3.0–4.9	0.062	OTH
**Piaggine**	Rural	1077	3	2.79	1.45	8.6–10.8	5.6–7.8	0.009	UD
**Pisciotta**	Rural	2404	2	0.83	0.59	19.2–24.0	17.2–22.0	<0.001	UD
**Polla**	Rural	5078	2	0.39	0.34	40.6–50.8	38.6–48.8	<0.001	UD
**Pollica**	Rural	2102	2	0.95	0.65	16.8–21.0	14.8–19.0	<0.001	UD
**Positano**	Rural	3678	1	0.27	0.22	29.4–36.8	28.4–35.8	<0.001	UD
**Postiglione**	Rural	1998	3	1.50	1.01	16.0–20.0	13.0–17.0	<0.001	UD
**Prignano Cilento**	Rural	1081	4	3.70	1.93	8.6–10.8	4.6–6.8	0.022	UD
**Ravello**	Rural	2332	2	0.86	0.61	18.7–23.3	16.7–21.3	<0.001	UD
**Ricigliano**	Rural	1064	2	1.88	0.97	8.5–10.6	6.5–8.6	0.003	UD
**Roccadaspide**	Rural	6880	11	1.60	1.40	55.0–68.8	44.0–57.8	<0.001	UD
**Roccagloriosa**	Rural	1551	4	2.58	1.57	12.4–15.5	8.4–11.5	0.001	UD
**Romagnano al Monte**	Rural	378	1	2.65	0.73	3.0–3.8	2.0–2.8	0.113	OTH
**Roscigno**	Rural	579	2	3.45	1.27	4.6–5.8	2.6–3.8	0.074	OTH
**Rutino**	Rural	750	2	2.67	1.15	6.0–7.5	4.0–5.5	0.027	UD
**Sacco**	Rural	409	1	2.44	0.71	3.3–4.1	2.3–3.1	0.093	OTH
**Salento**	Rural	1744	22	12.61	8.02	14.0–17.4	−8.0–4.6	0.028	HS
**Salvitelle**	Rural	467	2	4.28	1.37	3.7–4.7	1.7–2.7	0.132	OTH
**San Giovanni a Piro**	Rural	3571	12	3.36	2.63	28.6–35.7	16.6–23.7	<0.001	UD
**San Gregorio Magno**	Rural	3866	2	0.52	0.42	30.9–38.7	28.9–36.7	<0.001	UD
**San Mango Piemonte**	Rural	2660	5	1.88	1.37	21.3–26.6	16.3–21.6	<0.001	UD
**San Pietro al Tanagro**	Rural	1669	3	1.80	1.13	13.4–16.7	10.4–13.7	<0.001	UD
**San Rufo**	Rural	1591	1	0.63	0.39	12.7–15.9	11.7–14.9	<0.001	UD
**Sant’Angelo a Fasanella**	Rural	500	1	2.00	0.67	4.0–5.0	3.0–4.0	0.050	OTH
**Sant’Arsenio**	Rural	2645	2	0.76	0.56	21.2–26.5	19.2–24.5	<0.001	UD
**Santa Marina**	Rural	3219	8	2.49	1.90	25.8–32.2	17.8–24.2	<0.001	UD
**Sanza**	Rural	2330	1	0.43	0.31	18.6–23.3	17.6–22.3	<0.001	UD
**Sassano**	Rural	4666	2	0.43	0.36	37.3–46.7	35.3–44.7	<0.001	UD
**Scala**	Rural	1504	3	1.99	1.20	12.0–15.0	9.0–12.0	<0.001	UD
**Serre**	Rural	3646	12	3.29	2.59	29.2–36.5	17.2–24.5	<0.001	UD
**Sessa Cilento**	Rural	1129	3	2.66	1.41	9.0–11.3	6.0–8.3	0.007	UD
**Sicignano degli Alburni**	Rural	3172	3	0.95	0.73	25.4–31.7	22.4–28.7	<0.001	UD
**Teggiano**	Rural	6993	4	0.57	0.51	55.9–69.9	51.9–65.9	<0.001	UD
**Torchiara**	Rural	1903	5	2.63	1.73	15.2–19.0	10.2–14.0	<0.001	UD
**Torraca**	Rural	1229	3	2.44	1.35	9.8–12.3	6.8–9.3	0.004	UD
**Torre Orsaia**	Rural	1938	2	1.03	0.69	15.5–19.4	13.5–17.4	<0.001	UD
**Tramonti**	Rural	4184	9	2.15	1.74	33.5–41.8	24.5–32.8	<0.001	UD
**Trentinara**	Rural	1561	1	0.64	0.40	12.5–15.6	11.5–14.6	<0.001	UD
**Valva**	Rural	1534	2	1.30	0.79	12.3–15.3	10.3–13.3	<0.001	UD
**Vibonati**	Rural	3205	5	1.56	1.20	25.6–32.1	20.6–27.1	<0.001	UD

The table summarizes the number of diagnosed and linked-to-care hepatitis C cases, along the observed prevalence per 1000 inhabitants, across various municipalities in the Province of Salerno. Expected cases were estimated by applying benchmark prevalence rates to the resident population of each municipality, using ranges of 0.6–0.8% for urban areas and 0.8–1.0% for rural areas. Bayesian prevalence per 1000 was calculated incorporating prior rates of 7 per 1000 (urban) and 9 per 1000 (rural)–selected as representative values within the respective benchmark ranges–and stabilization parameter. *P*-values were calculated using a Poisson test comparing observed vs. expected case counts. MUN: Municipality, POP: Population, OBS_C: Observed Cases, OBS_P: Observed Prevalence (per 1000), BAY_P: Bayesian Prevalence (per 1000), EXP_Min–Max: Expected cases, minimum-maximum, HB_Min–Max: Hidden Burden, minimum-maximum, CLS: Classification, UD: Underdiagnosed, HS: Hotspot, OTH: Other.

**Table 4:** Age-Specific Prevalence and Hidden Burden.

Age Group	Population	Observed Cases	Observed Prevalence Per 1000	Expected Prevalence %	Expected Cases	Hidden Burden	*p*-Value
**0–29 years**	294,967	38	0.13	0.12	354	316	<0.001
**30–45 years**	203,548	414	2.03	0.6	1221	807	<0.001
**46–56 years**	171,274	766	4.47	1.2	2055	1289	<0.001
**>56 years**	368,939	2310	6.26	2.5	9223	6913	<0.001
**Total**	1,038,728	3528	3.40	0.72	7479	3951	<0.001

**Table 5:** Logistic regression analysis of factors associated with advanced fibrosis and cirrhosis.

Variable	OR	95% CI	*p*-Value
Age group			
0–29 years	1 ^a^		
30–45 years	1.28	0.590–3.07	0.56
46–56 years	2.93	1.38–6.95	0.008
>56 years	4.50	2.14–10.6	<0.001
Sex			
Female	1 ^a^		
Male	1.63	1.42–1.89	<0.001
Urban/rural			
Rural	1 ^a^		
Urban	1.29	1.05–1.58	0.01
Genotype			
Others	1 ^a^		
1b	1.56	1.35–1.80	<0.001

Factors associated with advanced disease (F3–F4 vs. F0–F2) in HCV patients (Sample size = 3528). Log-likelihood ratio (G-squared) = 221, *p* < 0.001. 1 ^a^: reference category. Odds ratios (ORs) are reported with corresponding 95% confidence intervals (CIs) and *p*-values.

**Table 6:** Temporal Analysis of HCV Cases (2015–2018 vs. 2019–2022).

Parameter	2015–2018	2019–2022	*p*-Value
Expected HCV cases (period start)	7479	5237	<0.001 *
Observed/linked-to-care cases	2242	1286	
Hidden burden (period end)	5237	3951	
Mean Age (years) ± SD	63.2 (13.4)	61.9 (15.0)	0.02
F3–F4 fibrosis (%)	1363 (60.8%)	408 (31.7%)	<0.001
Urban residence (%)	1955 (87.2%)	1112 (86.5%)	0.5362

* Refers to both the Poisson test results in the two different periods, comparing observed and expected cases.

## Data Availability

The data presented in this study are available from the corresponding author upon request.

## Abbreviations

The following abbreviations are used in this manuscript:

HCV	Hepatitis C Virus
WHO	World Health Organization
PWID	People who inject drugs
MSM	Men who have sex with men
GPs	General practitioners
DAAs	Direct-acting antivirals
ISTAT	Istituto nazionale di statistica
SVR	Sustained virologic response
VCTE	Vibration-controlled transient elastography
OECD	Organisation for Economic Co-operation and Development
Eurostat	European Statistical Office
SEIEVA	Sistema epidemiologico integrato delle epatiti virali acute
SD	Standard deviation
MUN	Municipality
POP	Population
OBS_C	Observed Cases
OBS_P	Observed Prevalence
BAY_P	Bayesian Prevalence
EXP_Min-Max	Expected cases, minimum–maximum
HB_Min-Max	Hidden Burden, minimum–maximum
CLS	Classification
UD	Underdiagnosed
HS	Hotspots
OTH	Other
QGIS	Quantum Geographic Information System
ORs	Odds ratios
CIs	Confidence intervals
IDU	Injecting drug use
MASLD	Metabolic dysfunction-associated steatotic liver disease
ECHO	Extension for Community Healthcare Outcomes
